# Whole-transcriptome RNA sequencing reveals global expression dynamics and ceRNA regulatory networks related to hair follicle development and melanogenesis in goats

**DOI:** 10.5713/ab.24.0617

**Published:** 2025-03-31

**Authors:** Junyin Zhao, Jipan Zhang, Ziyi Chen, Min Xiao, Yongju Zhao

**Affiliations:** 1 College of Animal Science and Technology, Southwest University, Chongqing Key Laboratory of Herbivore Science, Chongqing, China

**Keywords:** ceRNA, Coat Color, Goat, Hair Follicle, Whole Transcriptome

## Abstract

**Objective:**

Domestic animals, fur is a product of long-term selection by humans and the natural environment. It is generally used to distinguish between different breeds. This study aims to dissect the molecular mechanisms underlying the distinct fur characteristics of goats, particularly focusing on the molecular and regulatory differences between the Dazu Black Goat (DBG) and the Inner Mongolia Cashmere Goat (IMCG). Through whole-transcriptome analysis, we aim to identify differentially expressed RNAs and construct a ceRNA network to reveal the genetic regulation of goat hair follicle development and melanin production.

**Methods:**

Skin, hair, and cashmere samples were collected from DBG (n = 15) and IMCG (n = 17) to assess hair follicle density, length, diameter, and melanin content. Whole-transcriptome sequencing of skin tissues from DBG (n = 3) and IMCG (n = 3) identified 50,652 RNAs. Differential expression analysis was performed on mRNAs, lncRNAs, miRNAs, and circRNAs.

**Results:**

IMCG exhibited significantly higher hair follicle density, hair length, and cashmere diameter than DBG (p < 0.01), whereas DBG had significantly thicker hair and higher melanin content (p < 0.01). A total of 640 differentially expressed RNAs were identified, including 157 mRNAs, 234 lncRNAs, 72 miRNAs, and 177 circRNAs. These were enriched in pathways related to melanogenesis, hair follicle development, and GO terms such as collagen fiber organization and pigmentation. ceRNA networks constructed from differentially expressed RNAs revealed key regulatory mechanisms of coat color and hair traits.

**Conclusion:**

Whole-transcriptome sequencing revealed expression profiles and ceRNA networks involved in hair follicle development and melanogenesis in goats. These findings provide insights into the roles of coding and non-coding RNAs in fur traits, supporting future breeding strategies and textile applications.

## INTRODUCTION

The Inner Mongolia cashmere goat (IMCG) and the Dazu black goat (DBG) are two representative cashmere- and meat-goat breeds from China [1,2]. The cashmere fiber and coat color of goats have been widely studied in recent years [1,3], and the diverse assortment of coat colors in livestock arises from deliberate human selection, underscoring its essential role in defining distinctive breed identities [4,5]. As a domesticated animal, the fur color phenotype of goats has been extensively studied, representing one of the focal points of research. The coat color of goats is typically characterized by a consistent and predominantly homogeneous appearance, exhibiting remarkable purity. The hair follicle characteristics of goats exert a direct and crucial influence on both the quantity and quality of cashmere fibers produced, where hair follicle development is governed by a multitude of intricate mechanisms, involving regulatory processes at various levels, including transcription and translation [6]. Cashmere mainly derives from secondary hair follicles (SHFs), characterized by their soft and comfortable texture, while wool primarily originates from primary hair follicles, which are generally thicker and longer, playing a protective role for the goats [7]. Although melanin deposition and hair follicle development in goats are important and complex processes, their underlying mechanisms and molecular modes of action are not fully understood.

Recent studies on coat color and hair follicle development have produced compelling evidence that highlights the intricate interplay between messenger RNAs (mRNAs) and non-coding RNAs (ncRNAs) [8,9]. The miRNAs, measuring approximately 22 nucleotides in length, regulate gene expression post-transcriptionally by primarily binding to the 3′ untranslated region of target mRNAs [10]. Some miRNA studies on hair follicle development and coat color have shown that miRNA-203 may regulate hair follicle development in cashmere goats by targeting the *DDOST* and *NAE1* [11], while miR-143-3p directly inhibits the expression of Itga6, playing a crucial role in cashmere growth [12]. Similarly, miRNA-101a-3p and miRNA-144a-3p regulate critical genes associated with melanocytosis in alpaca melanocytes [13]. Also, overexpression of miRNA-27a-3p inhibits the expression of mRNA and protein related to melanogenesis, thereby reducing the melanin content of human epidermal melanocytes [14].

Long non-coding RNAs (lncRNAs) are defined as ncRNAs exceeding 200 nucleotides, participating in the regulation of gene expression and various cellular processes [15]. In the Jiangnan cashmere goat, LncRNA-599554 promotes Wnt3a expression by sequestering miR-15a-5p, thereby enhancing the inductive capability of dermal papilla cells [16]. Additionally, lncRNA SPRIGHTLY regulates the proliferation of cells and anchorage-independent colony formation in human primary melanocytes [17]. A network analysis of lncRNA-mRNA interactions in the skin of Liaoning cashmere goats and Ziwuling black goats revealed the presence of 22 pairs of trans-target genes associated with seven differentially expressed lncRNAs. Among these, 13 trans-target genes were found to be involved in the regulation of cashmere fiber diameter, while nine trans-target genes were responsible for the cashmere fiber color [18].

Circular RNAs (circRNAs), representing a novel class of RNA molecules, possess remarkably stable structures characterized by a covalently closed loop lacking 5′ and 3′ free ends [19]. The pivotal role of circRNAs in gene regulation has been increasingly reported [20]. Nevertheless, the functional implications of circRNAs concerning goat hair follicle development and melanogenesis remain poorly studied. It is, however, known that CircRNA-0100 acts as a positive regulator in the differentiation of cashmere goat SHF-stem cells (SCs) into the hair follicle lineage by sequestering miR-153-3p, resulting in the elevation of *KLF5* expression [21]. Also, CircRNA-1926 facilitates the differentiation of goat SHF-SCs into the hair follicle lineage through the miR-148a/b-3p/CDK19 axis [22]. The participation of the Circ0091223/miR-1291/TYR ceRNA network in α-MSH-induced melanogenesis highlights its potential as a promising target for therapeutic interventions [23]. Competing endogenous RNAs (ceRNAs) regulate miRNA target gene expression by binding miRNAs through their response elements[24]. Evidence suggests that ceRNAs play significant roles in various biological processes in vertebrates [25,26]. The ceRNA network of lncRNA and circRNA has complex interactions involving ncRNA and mRNA related to goat skin and melanin deposition. However, the role of these ncRNAs is poorly understood.

The development of hair follicles and melanogenesis in goats are highly intricate biological processes. In this study, to investigate the regulatory mechanisms of goat hair follicle development and melanogenesis, we hypothesize that mRNA, lncRNA, miRNA, and circRNA regulate this process through enrichment in melanin and hair follicle development related pathways, as well as ncRNA-mRNA interactions. To verify this hypothesis, we compared the whole-transcriptome expression profiles of these RNA molecules in DBG and IMCG skin tissues. And Gene Ontology (GO) and Kyoto Encyclopedia of Genes and Genomes (KEGG) enrichment analysis were conducted on the differentially expressed RNA parent genes to screen for the affected biological pathways and functions. In addition, we constructed and analyzed a co-expression network of miRNA, circRNA, lncRNA, and mRNA to reveal potential ceRNA interactions during hair follicle development and melanogenesis. The results of this study provide a basis for us to understand the complex molecular mechanisms of skin physiology and melanogenesis, which will also expand our understanding of the diversity of coat color in different domestic animals.

## MATERIALS AND METHODS

### Animals and samples

All experimental procedures were approved by the Animal Ethics Committee of Southwest University (Ethics approval No. LAC2023-1-0147). This work includes 17 adult IMCGs and 15 adult DBGs. Of these, 11 IMCGs (five males and six females, selected from a private farm in Baotou city, Inner Mongolia Autonomous Region, China) and nine DBGs (four males and five females, selected from Tengda farm in Dazu district, Chongqing, China) were slaughtered by jugular vein bloodletting. From each goat, the fleece fiber and skin tissue from the lateral chest were collected. The skin tissues were stored in a 5 mL-centrifugal tube with approximately 3 mL of 10% formaldehyde for paraffin sectioning and hematoxylin and eosin (H&E) staining.

The other 12 goats, including six female IMCGs and six female DBGs, were housed on the breeding farm of Southwest University, Chongqing, China. Skin tissue was sampled as follows: (1) Skin fibers from a 5 cm×5 cm area of the lateral chest were shed by an electric hair clipper (FC5902; Flyco, Shanghai, China). (2) Goats were anesthetized through intramuscular injection of xylazine hydrochloride (#180121777; Baite, Changsha, China), at a concentration of 0.2 mg per kg of body weight. (3) Goats were transferred to an operating table, and the exposed skin was swabbed with alcohol to disinfect it. (4) The skin tissue, approximately 1 cm^2^, was grasped with sterile forceps and quickly cut near the tip using sterile scalpel blades. (5) The surgical wound was stitched with a suture and sterilized with iodophor. (6) Then, idazoxan hydrochloride (#180122315, Baite), was injected at a concentration of 0.2 mg per kg body weight. (7) After the surgical procedure, animals were returned to the pen and were carefully monitored during the two weeks after surgery. Skin samples were immediately stored in liquid nitrogen, then transferred to the laboratory and stored at −80°C for whole transcriptome sequencing and quantitative polymerase chain reaction (qPCR) experiments.

### Skin follicle density, fiber characteristics, and melanin content

After 48 h fixation, the skin samples were processed as follows: dehydration, embedding, sectioning, H&E staining, and slice sealing. One high-quality image (9.26 mm^2^) was captured for each stained section using an inverted microscope (IX51; Olympus, Tokyo, Japan). All the primary and SHFs were manually marked for each original image using the Image-Pro Plus 6.0 software, where a self-written Matlab script was used to calculate total hair follicle density.

Then, cashmere length and hair length were measured with a steel ruler. The fleece sample was washed with absolute ethanol thrice and oven-dried at 50°C for 6 h. The hair fibers were cut into 1 to 2 mm lengths in a Petri dish, and then placed on a glass slide. A drop of glycerol was added, and the sample was covered with a coverslip. Under the inverted microscope, the Cellsens software was used to measure the cashmere diameter and hair diameter of each sample 30 times.

The relative melanin content of each fleece sample was measured with a NaOH assay. Briefly, 15 to 30 mg of each fleece sample was put into a 5 mL centrifuge tube containing 1 mL of 1 mol/L NaOH and bathed in water at 95 degrees for 1 h. The absorbance was measured at 500nm using a microplate reader (BIO-RAD, Hercules, CA, USA). The working curve was established based on the absorbance of standard melanin at different concentrations, including 0, 0.02, 0.04, 0.06, 0.08, 0.1, 0.12, 0.14, 0.16, 0.2, 0.3, 0.4, 0.5, 0.6 mg/mL, to calculate the melanin content of the hair samples.

### RNA extraction and transcriptome sequencing

The sequencing strategy involves collecting skin samples from two goats of the same breed to form a library, generating six libraries from 12 goats. TRIzol Reagent (lncRNA) was used to extract total RNA (including mRNA, lncRNA, and cyclic RNA) from each skin sample following the manufacturer’s protocol. A Nanodrop 8000 Spectrophotometer (NanoDrop Technologies, Wilmington, DE, USA) was used to detect the quality of the RNA, and an equal amount of RNA from each skin group was pooled for library preparation. The central ribosome zero ribosomal RNA (rRNA) removal kit (Central, Madison, WI, USA) was used to remove rRNA, and subsequently no rRNA residues were precipitated with ethanol. The RNA library preparation kit of NEBNext directed Illumina (NEB, Ipswich, MA, USA) was used to generate High-strand-specificity libraries from each pooled sample.

Library quality testing was performed using the DNA 1000 testing kit (Agilent Technologies, Santa Clara, CA, USA) or the High-Sensitivity DNA testing kit (Agilent Technologies). The ABI StepOnePlus real-time PCR system (Life Technologies, Carlsbad, CA, USA) was used for quantitative analysis. Transcriptome sequencing was performed using the Hiseq2500 and PE150 platforms. Subsequently, PCR products were purified using the AMPure XP system, and the library quality was evaluated using the Agilent Bioanalyzer 2100 and qPCR methods.

### Quality control of raw reads and differential expression analysis

All raw reads were first processed through in-house Perl scripts and clean data (clean reads) were obtained by removing reads containing adapters, ploy-N segments, and low-quality reads from raw data. At the same time, Q20, Q30, guanine-cytosine-content and sequence duplication levels of the clean data were calculated. All the downstream analyses were based on clean data with high quality. The DESeq2 R package (version 1.22.2) was utilized to pinpoint differentially expressed transcripts, normalizing read counts and applying the negative binomial distribution to model gene expression variance. Adjustments for multiple testing were made using the Benjamini-Hochberg method, with a significance threshold set at p<0.05. The log2 fold change was calculated to assess expression differences, leading to the identification of mRNAs, lncRNAs, miRNAs, and circRNAs with significant expression variations between DBG and IMCG skin tissues, thereby elucidating the molecular basis of their hair phenotypes.

### LncRNA identification

The StringTie (v1.3.1) software was used to identify and quantify lncRNAs and coding genes by calculating fragments per kilobase of exon per million fragments mapped (FPKM) values for each transcript. FPKM is determined based on the length of the RNA fragments and the number of reads mapped to each transcript. For each sample, both lncRNAs and coding genes were assessed. Gene FPKM was calculated by summing the FPKM values of all the transcripts within a specific gene group, which accounts for the expression levels of individual isoforms within the gene. To improve the accuracy of lncRNA identification, we used a combination of the transcript annotation from StringTie and comparison to known lncRNA databases, ensuring that only high-confidence lncRNAs were retained for further analysis. Additionally, potential lncRNAs were further filtered based on length (>200 bp) and exon number, while non-coding potential was confirmed using the Coding Potential Calculator and Pfam databases.

### miRNA Identification and target-gene prediction

The GeneBank (release 209.0) and Rfam (11.0) databases were utilized for data annotation, enabling the efficient removal of rRNAs, scRNAs, snoRNAs, snRNAs, and tRNAs from the small RNA (sRNA) dataset. TargetScan (version 7.0) and miRanda (version 3.3a) software were used to predict the target genes of skin tissue miRNAs. The intersecting results were considered high-confidence predictions for miRNA target genes. To assess the expression correlation, we used the Spearman rank correlation coefficient (SCC), selecting pairs with SCC<−0.7 as negatively coexpressed mRNA–miRNA pairs, where the mRNAs were the target genes of DEmiRNAs.

### CircRNA identification and source-gene prediction

The goat genome (ARS1) served as the reference panel for sequence alignment and subsequent analysis. Then, clean reads were aligned with the reference genome to obtain positional information on the reference genome and genes. Then, reverse splicing algorithms were used to identify unmapped reads. Using Burrows-Wheeler-Alignment (BWA) for comparison, we referred to the reads mapped to the reference genome as Mapped Reads, and the corresponding data as Mapped Data. Sequencing reads and reference genome sequence alignment result files (BAM format), species reference genome sequences, and annotation files visually browsed using the Integrated Genomics Viewer. The CIRI software was used to predict CircRNA from scratch. The CIRI software used the BWA software to compare goat reference gene sequences, generate a Sequence Alignment/MAP format (SAM) file, and to analyze the Concise Idiosyncratic Gapped Alignment Report (CIGAR) values in the SAM file. The paid chiastic clipping (PCC) signals were scanned from the SAM file and the characteristics of the CIGAR value in the junction read was: xS/HyM or xMyS/H, where x and y represent the number of bases, M is mapping, S is soft clipping, and H is hard clipping. For dual ended reads, the CIRI algorithm considers a pair of reads, one of which can be mapped to the CircRNA and the other needs to be mapped to the range of the CircRNA. For the circular structure formed by the single exon formation or “long exon 1-short exon long exon 2”, the CIGAR value should be xS/HyMzS/H and (x+y) S/HzM or xM (y+z) S/H, where the CIRI software can separate these two situations. For splicing signals (GT, AG), CIRI also considers other weak splicing information, such as (AT-AC). The algorithm extracts exon boundary positions from the GTF/GFF file and filters false positives using a known range.

### Reverse-transcription quantitative polymerase chain reaction verification

The samples used for the qPCR analyses were the same as those used in the RNA sequencing (RNA-seq) study. The cDNA synthesis for the mRNA, lncRNA, and circRNA samples was executed using the PrimeScript RT reagent kit with gDNA Eraser (Takara, Dalian, China). For the miRNA, reverse transcription was performed using the Mir-X miRNA First-Strand Synthesis kit (TaKaRa). Primer sequences for the mRNAs and ncRNAs were meticulously designed and synthesized by primer primer 5, as outlined in the Supplement 1. The *β-actin* gene served as an internal control for the mRNAs, lncRNAs and circRNAs, while the *U6* gene served as an internal control for the miRNAs. Relative quantification of the results was performed using the 2^−ΔΔCT^ method.

### Statistical analysis

All non-sequencing data were expressed as the mean value ± standard deviation. Before proceeding with the analysis, we conducted tests for normality (Shapiro–Wilk test) and homogeneity of variance (Levene’s test) on all data sets to ensure their suitability for subsequent statistical procedures.Statistical analysis was conducted using SPSS 18.0, and an independent t-test was used for comparison between two groups. The level of significance was set as * p<0.05, ** p<0.01. Co-expression networks involving miRNA-mRNA, lncRNA-miRNA-mRNA, and circRNA-miRNA-mRNA were meticulously constructed using the Cytoscape software (v3.10.0; Cytoscape Consortium, San Diego, CA, USA). CeRNAs were selected based on the criteria that they shared a number of miRNAs greater than 3, had a hypergeometric test p-value less than 0.05, and an adjusted false discovery rate value less than 0.05 for differential genes. Pairs with a PCC greater than 0.8 were identified as co-expressed circRNA/lncRNA-miRNA-mRNA pairs.

## RESULTS

### Phenotypic characteristics of Dazu black goat and Inner Mongolia cashmere goat

**Figure 1:**
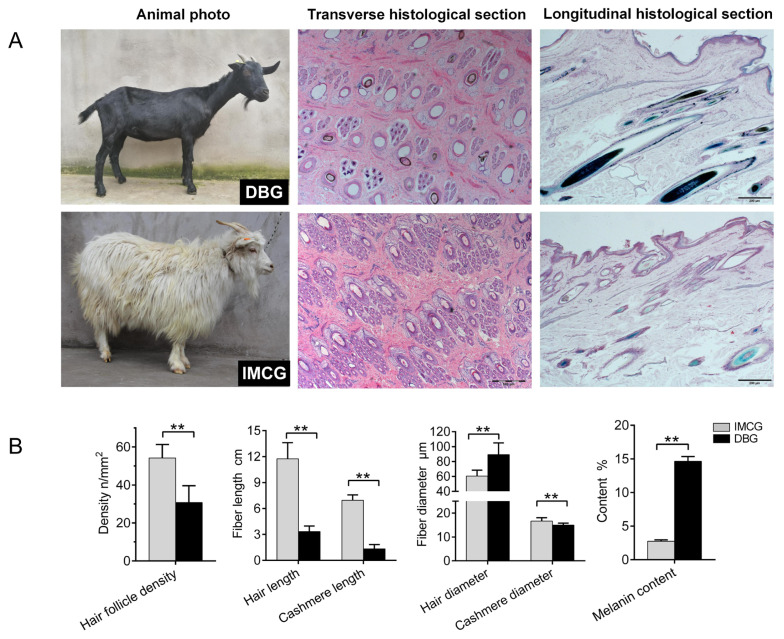
Skin phenotype indicators of two types of goats. (A) The phenotypic differences in animal individual, skin surface, skin histolgical section between DBG and IMCG. (B) The hair follicle and fiber perimeter of DBG and IMCG. The data are expressed as mean values±standard deviations, and ** significant differences (p<0.01) as determined by t-test. DBG, Dazu black goat; IMCG, Inner Mongolia cashmere goat.

The whole body of the DBG is covered with pure black and shiny fur, with short and tight hair strands that adhere to the skin; the IMCGs are pure white, with high-quality cashmere (Figure 1A). HE staining of the skin cross-sections and melanin staining of the skin longitudinal sections of both DBG and IMCG vividly depicted hair follicle density and melanin deposition (Figure 1A). We conducted a statistical analysis of the six primary indicators related to goat hair, where hair follicle density, hair length, and cashmere diameter were significantly higher in IMCG (p<0.01) compared to DBG (Figure 1B). Conversely, DBG exhibited significantly thicker hair diameter and more melanin content than IMCG (p<0.01).

### Identification of RNAs in Dazu black goat and Inner Mongolia cashmere goat skin by RNA-sequencing

**Figure 2:**
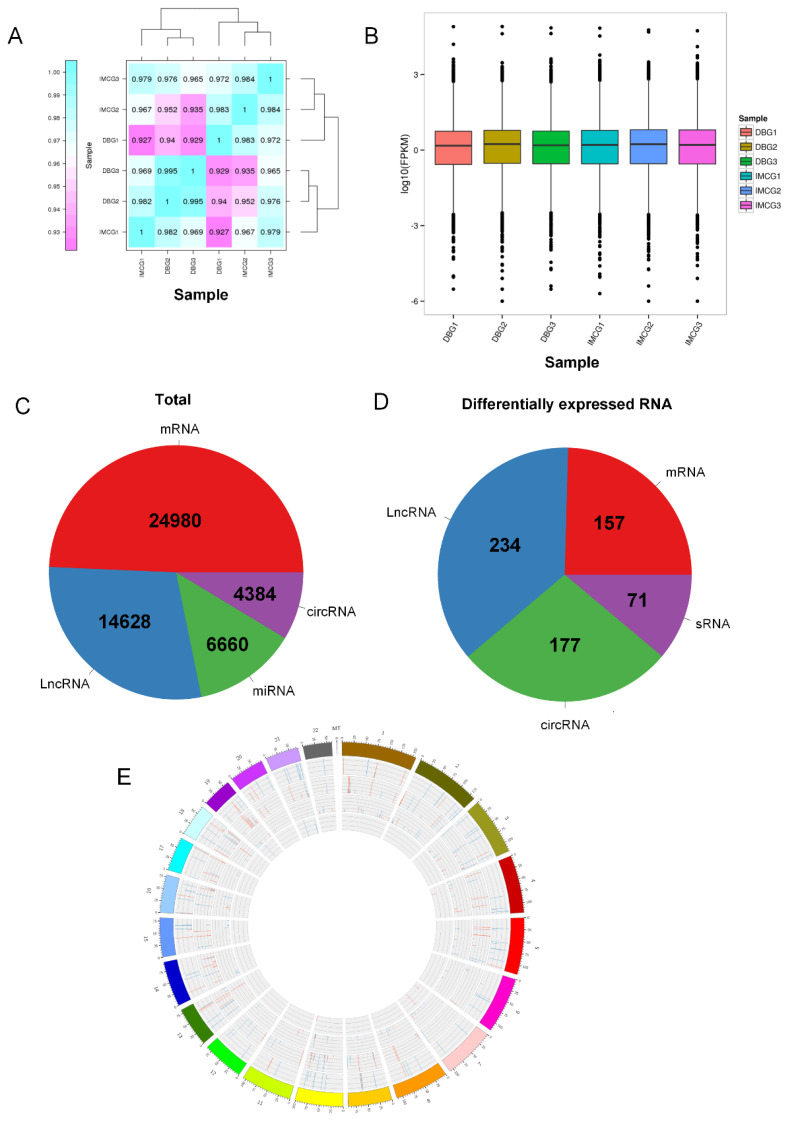
Distribution of total RNA and differential RNA. (A) Correlation analysis matrix diagram. (B) Boxplot of all RNA expression levels. (C) Proportion distribution of total RNA. (D) Differential RNA distribution. (E) Circos diagram of chromosome distribution of DERNAs.

The FASTQ data obtained through the high-throughput sequencing was used to predict and identify various RNAs. The number of these RNAs was quantified in conjunction with known RNAs. The skin tissues of DBG and IMCG were divided into six groups and a total of six lncRNA and mRNA libraries, six circRNA libraries, and six miRNA libraries were sequenced on the Illumina HiSeqTM 2500 platform and underwent preliminary filtering, resulting in approximately 94.32GB of clean lncRNA sequence data and 94.31GB of clean circRNA data. The miRNAs reads were cleaned by removing the sequences shorter than 18nt or longer than 30nt. Finally, 147621953 clear reads of sRNA were obtained. Simultaneously, the Q30 of each lncRNA library was above 94.17%, and the Q30 of each circRNA library was above 97.96% (Supplement 2). The correlation between DBG and IMCG inter-group and intra-group relationship samples was good (Figures 2A, 2B). A total of 50,652 RNAs were identified in this study, including 24,980 mRNAs, 14,628 lncRNAs, 4,384 circRNAs, and 6,660 miRNAs (Figure 2C). The chromosome distribution of DEmRNAs is shown in the Circos plot (Figure 2E).

### Transcriptional profiling of DEmRNAs

**Figure 3:**
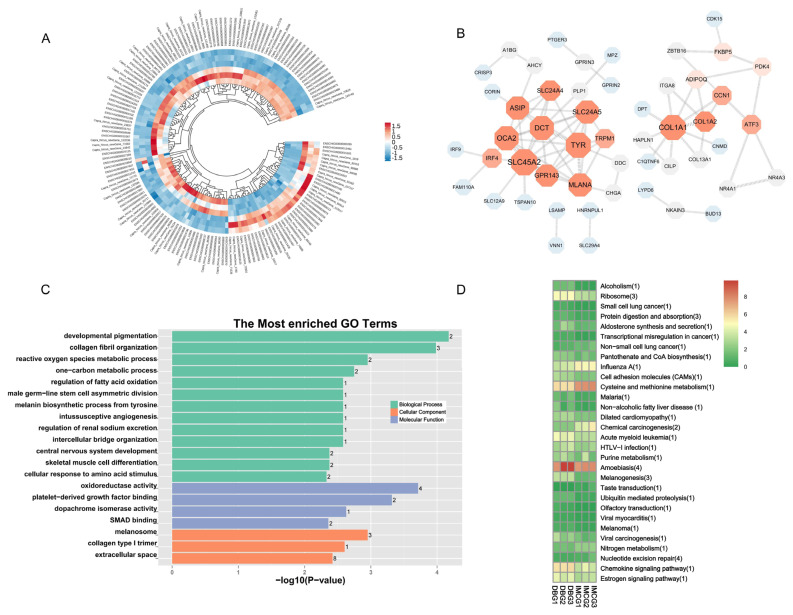
Functional analysis of DEmRNA in skin tissue of DBG and IMCG. (A) Dynamic circular heat map of DEmRNAs in skin tissue of DBG and IMCG. (B) PPI network analysis of the DEmRNAs. (C) Top20 terms of GO enrichment analysis. The number of differentially expressed genes at the end of the bar representation. (D) Top20 pathways of KEGG enrichment analysis. DBG, Dazu black goat; IMCG, Inner Mongolia cashmere goat; PPI, protein-protein interaction; GO, Gene Ontology; KEGG, Kyoto Encyclopedia of Genes and Genomes.

A total of 24,980 mRNAs were obtained, and 157 significantly different mRNAs were identified between the DBG and IMCG groups. Of these, 42 mRNAs were upregulated and 115 were downregulated from DBG to IMCG. Additionally, 234 differentially expressed lncRNAs (DElncRNAs) were identified between the DBG and IMCG groups. A circular heat map and protein-protein interaction network of the differentially expressed mRNAs (DEmRNAs) was generated to visualize DEmRNA expression patterns (Figures 3A, 3B). After comparing the DEmRNAs between groups, the GO function was assessed. The DEmRNAs were mainly enriched in developmental pigmentation, collagen fibril organization and reactive oxygen species metabolic processes in biological processes; oxidoreductase activity, SMAD binding, platelet-derived growth factor binding in cellular component; collagen type I trimer, and extracellular space in molecular function (Figure 3C). The result of the KEGG analysis indicated that the DEmRNAs were primarily annotated in the Amoebiasis, Melanogenesis, Protein digestion and absorption (Figure 3D).

### Expression patterns of DElncRNAs

**Figure 4:**
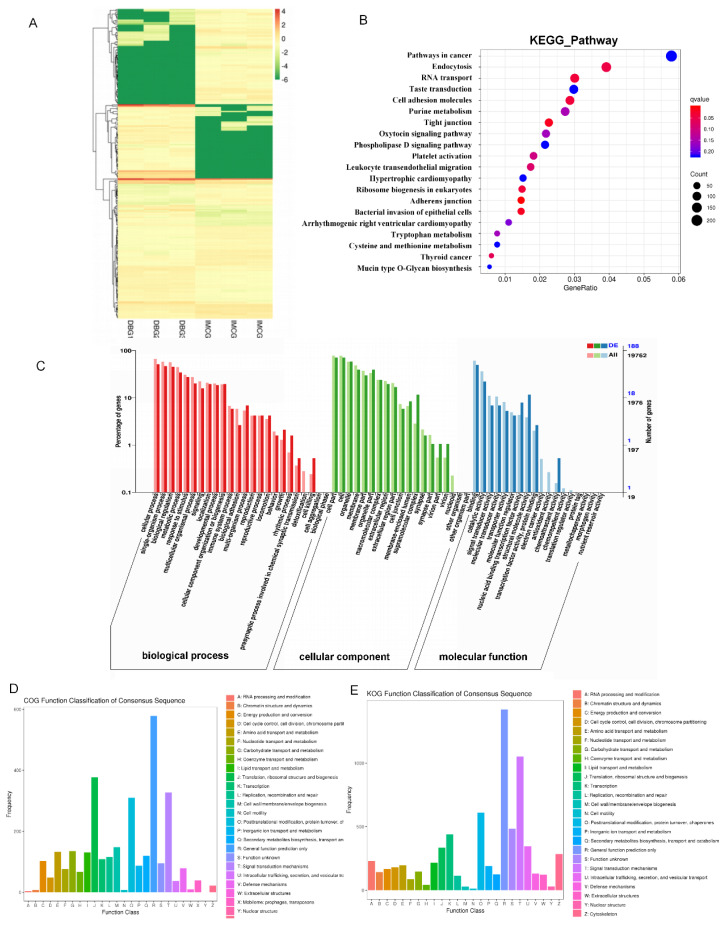
Expression profles of lncRNAs in the skin of DBG and IMCG. (A) Heat map of DElncRNAs. (B) Top20 pathways of KEGG enrichment analysis. (C) GO enrichment analysis of lncRNAs. (D) The KOG analysis of target genes of lncRNA. (E) The COG analysis of target genes of lncRNA. DBG, Dazu black goat; IMCG, Inner Mongolia cashmere goat; KEGG, Kyoto Encyclopedia of Genes and Genomes; GO, Gene Ontology; KOG, eukaryotic orthologous group; COGs, clusters of orthologous groups.

We identified 234 DElncRNAs of which 128 were upregulated and 106 were downregulated from DBG to IMCG (Figure 4A). The DElncRNA were primarily enriched in the GO terms related to cellular process, cell part and binding (Figure 4C). The KEGG analysis revealed that these DElncRNAs were significantly enriched (p<0.05) in pathways associated with the Pathways in cancer, Endocytosis, RNA transport and taste transduction (Figure 4B). Moreover, Tese protein clusters included general function prediction only, signal transduction mechanisms, translation, ribosomal structure and biogenesis, posttranslational modification, protein turnover, chaperones, secondary metabolite biosynthesis, transport and catabolism and cytoskeleton (Figures 4D, 4E).

### Identification of DEmiRNAs and functional annotation of target genes

**Figure 5:**
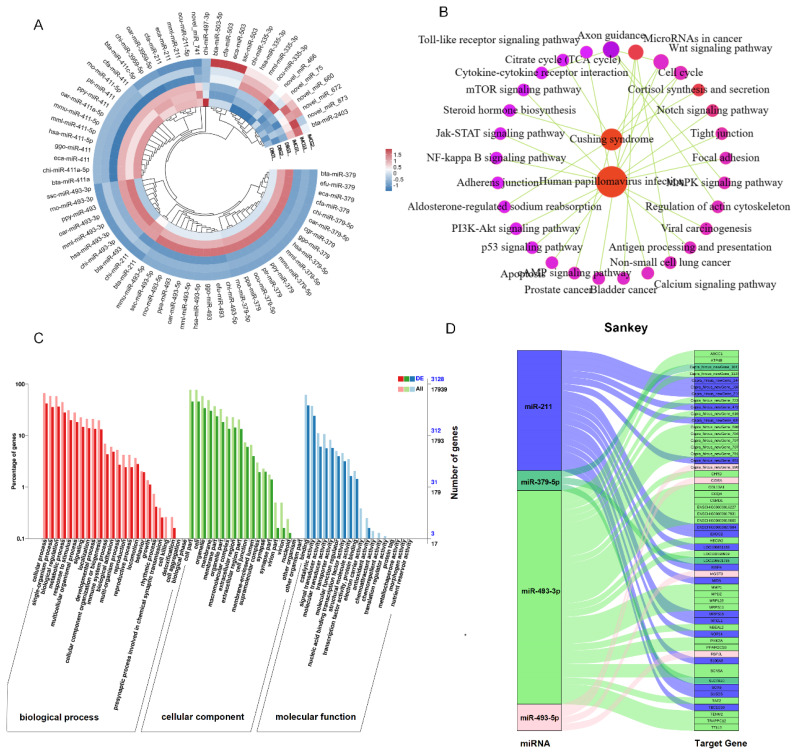
Functional analysis of DEmiRNAs between skin tissues of Dazu black goat and Inner Mongolia cashmere goat. (A) Heat map of 72 DEmiRNAs; red represents up-regulated and blue represents down-regulated. (B) KEGG_Pathway network diagram of target gene of DEmiRNAs. (C) GO terms of target genes of DEmiRNAs. (D) Sankey diagram of DEmiRNAs and target gene. KEGG, Kyoto Encyclopedia of Genes and Genomes; GO, Gene Ontology.

A total of 72 significant DEmiRNAs were identified between the DBG and IMCG groups, with 14 upregulated and 58 downregulated DEmiRNAs from DBG to IMCG (Figure 5A). Six sets of samples were filtered and the sequencing results were presented in the Supplement 3. The target genes of the DEmiRNAs were mainly enriched in the Wnt signaling pathway, the Jak-STAT signaling pathway, the Notch signaling pathway, and the MAPK signaling pathway (Figure 5B). Moreover, the target genes of the DEmiRNAs were mainly enriched in GO terms related to cellular processes, cell parts, binding, and developmental processes (Figure 5C). The most significant DEmiRNAs were identified and their target genes predicted, as shown in the Sankey diagram (Figure 5D).

### DEcircRNAs and functional annotation of DEcircRNA host genes

**Figure 6:**
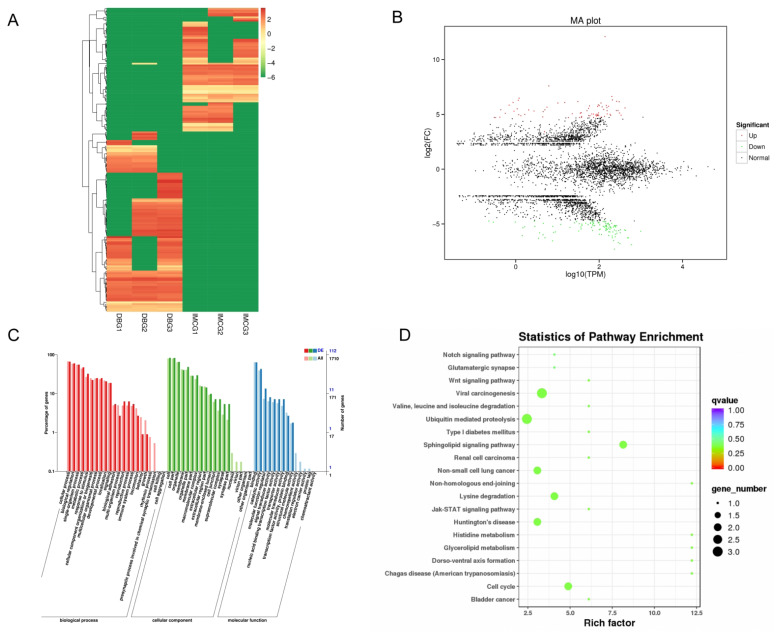
Functional analysis of DEcircRNAs in skin tissue of Dazu black goat and Inner Mongolia cashmere goat. (A) Heat map of 177 DEcircRNAs; red represents up-regulated and green represents down-regulated. (B) Volcano plot of DEcircRNA. (C)The most enriched GO terms of host genes of circRNAs in DBG and MCG. (D) KEGG pathways of the host genes. GO, Gene Ontology; KEGG, Kyoto Encyclopedia of Genes and Genomes.

A total of 177 DEcircRNAs were identified, including 72 up-regulated and 105 down-regulated from DBG to IMCG. The heatmap and volcano plot of the host genes of the DEcircRNA were hierarchically clustered (Figures 6A, 6B). Then, GO analysis revealed that 112 DEcircRNAs were enriched in 57 GO terms (Figure 6C) while 54 DEcircRNAs were enriched in 45 pathways. Interestingly, many of the top 20 enriched pathways originated from the Notch signaling pathway, the Wnt signaling pathway, the Jak-STAT signaling pathway, and the cell cycle (Figure 6D).

### Interaction network between non-coding RNA and mRNA

**Figure 7:**
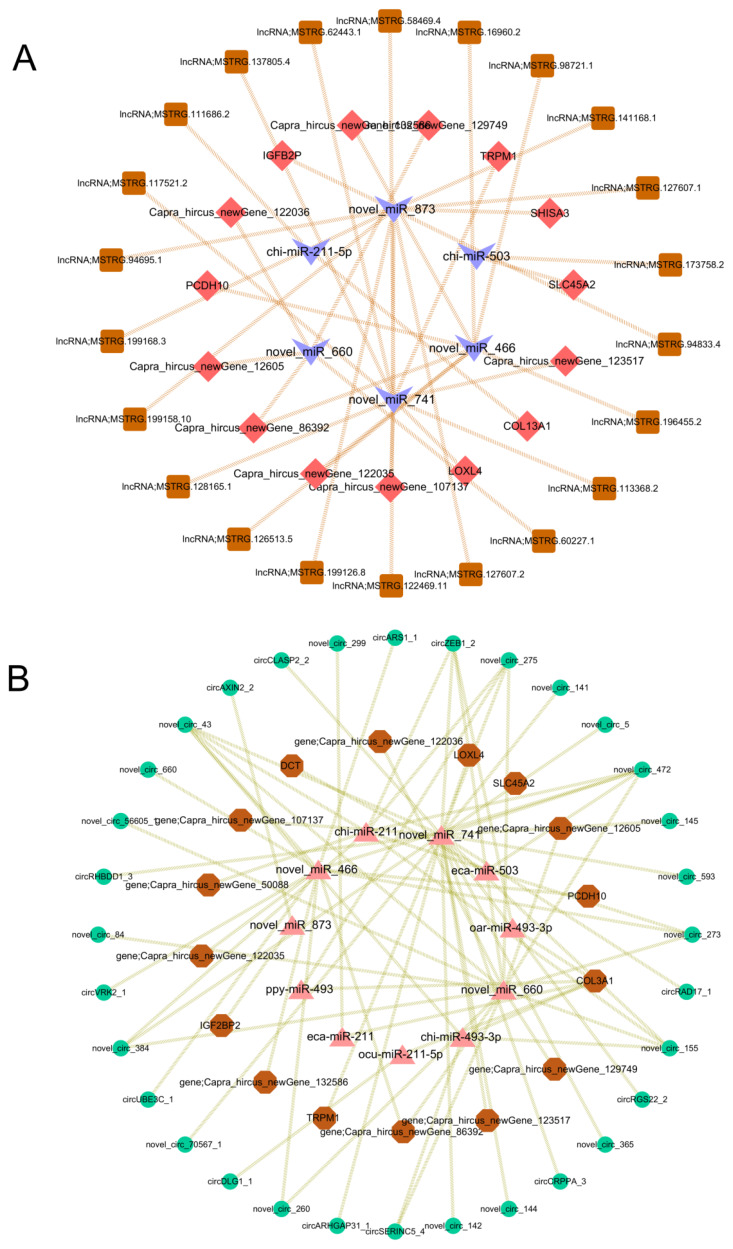
The co-expression network of lncRNA-miRNA-mRNA and circRNA-miRNA-mRNA. (A) LncRNA miRNA mRNA co expression network. Brown, light purple, pink represent lncRNA, miRNA, and mRNA, respectively. (B) circRNA-miRNA-mRNA co-expression network. Green, pink and brown represent circRNA, miRNA, and mRNA, respectively.

The DEncRNA-DEmRNA co-expression network was reconstructed in Cytoscape (v3.10.0). The DElncRNA-DEmiRNA-DEmRNA network was composed of 22 lncRNA nodes, 15 mRNA nodes, and 6 miRNA nodes (Figure 7A). The DEcircRNA-DEmiRNA-DEmRNA network was composed of 32 circRNA nodes, 19 mRNA nodes, and 9 miRNA nodes (Figure 7B). The relationships between the DERNAs could be viewed intuitively and clearly through the interaction network.

### Validation of differential expression RNAs by reverse transcription quantitative polymerase chain reaction

**Figure 8:**
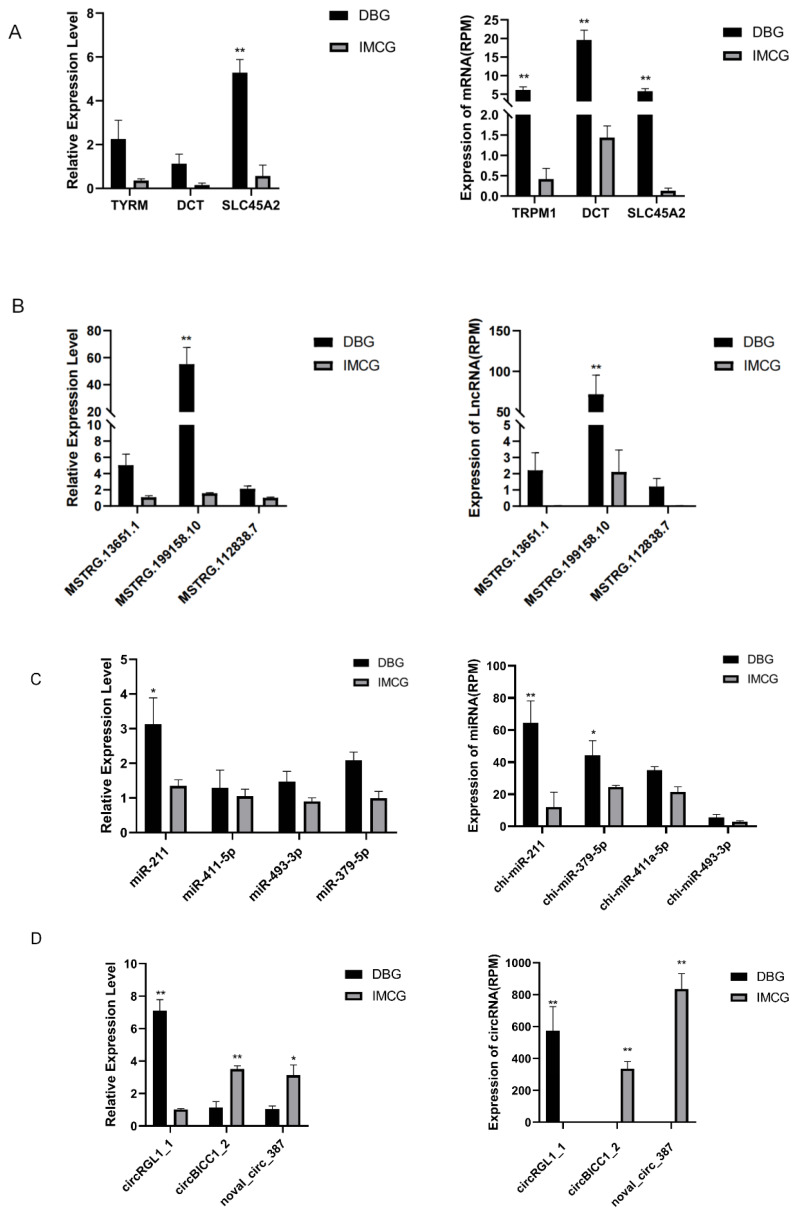
DEmRNAs, DElncRNAs, DEmiRNAs, and DEcircRNAs verified by RT-qPCR in skin of DBG and IMCG RNA-seq results. (A) Expression levels of mRNA by RNA-seq and RT-qPCR, (B) expression levels of lncRNA by RNA-seq and RT-qPCR, (C) expression levels of miRNA by RNA-seq and RT-qPCR, (D) expression levels of circRNA by RNA-seq and RT-qPCR. * p<0.05, ** p<0.01. qPCR, quantitative polymerase chain reaction; DBG, Dazu black goat; IMCG, Inner Mongolia cashmere goat; RT-qPCR, reverse transcription quantitative polymerase chain reaction.

Thirteen RNAs (3 mRNAs, 3 lncRNAs, 3 circRNAs, and 4 miRNAs) were randomly selected for verification using qRT-PCR, to confirm the accuracy of the RNA-seq results in this study (Figure 8). The results showed that through RNA seq, there were 3 mRNA, 1 lncRNA, 2 miRNAs, and 3 circRNAs between the DBG and IMCG groups, with significant differences. However, in the QPCR detection, only 1 mRNA, 1 lncRNA, 1 miRNA, and 3 circRNAs exhibited significant differences in both relative expression levels and differential expression. Figures 8A–8D present the relative expression levels of various RNAs as determined by both RNA-seq (represented on the right side of each panel) and RT-qPCR (represented on the left side of each panel). The relative expression levels of other RNAs demonstrated consistent trends compared to the RNA-seq results, indicating a strong correlation between the two methods. This consistency across independent techniques validates the reliability of the sequencing and analysis results of this transcriptome.

## DISCUSSION

Hair follicle development and pigmentation in the skin are complex multifactorial processes. This study analyzed the circRNAs, lncRNAs, miRNAs and mRNAs in the skin tissues of DBGs and IMCGs, and several interesting links between the different types of DERNAs were found. A number of current studies have shown that nc RNA plays an important role in the development of hair follicles and pigmentation. However, there are few reports on skin lncRNA in goats. For example, the expression of lncRNA-000133 in the anagen of cashmere goat SHF was significantly higher than that in the telogen, indicating that lncRNR-000133 may be involved in the reconstruction of SHF and the formation and growth of cashmere fibers [27]. Some skin lncRNAs were reported for their specificity and functional protection during skin development and pigmentation [28]. In this study, the target genes of some DElncRNAs were mainly enriched in Melanogenesis, the PI3K-Akt signaling pathway, as well as Protein digestion and absorption. Some other studies have shown that these pathways are involved in regulating coat color and hair follicle development [29].

The GO function analysis revealed that DEmRNAs mainly participated in developmental pigmentation, collagen fibril organization, collagen typeI trimer, and extracellular space. In addition, a large number of DEmRNAs were enriched in PI3K-Akt, ECM-receptor, and Melanogenesis, which are known to be associated with the development of cashmere hair follicles and pigmentation [30].

Similarly, studies have shown that circRNA can participate in the regulation of hair follicle development and pigmentation [21,31]. In this study, a large number of DEcircRNAs were identified, where their host genes were mainly enriched in Notch signaling pathway, Wnt signaling pathway, Jak-STAT signaling pathway, Sphingolipid signaling pathway, and Cell cycle. The Wnt signaling pathway leads to pigmentation and hair follicle development [32], while the Notch signaling pathway is involved in the regulation of melanin deposition and goat hair follicle development [33]. Furthermore, DEcircRNAs such as circ_RGL1_1 are specifically expressed in IMCGs, while others like circ_BICC1_2 are uniquely expressed in DBGs, suggesting that these circRNAs may play a significant role in hair follicle development and melanogenesis in goats. Our study also identified a substantial number of DEcircRNAs, indicating a potential close association between circRNAs and skin follicles in the fleece, with these DEcircRNAs likely participating in hair follicle development and melanogenesis through the modulation of relevant signaling pathways.

Numerous studies have shown that miRNA have specific expression patterns in cutaneous tissues, and play a key role in the periodic development of hair follicles and pigmentation [34]. For example, miR-193b is involved in regulating hair color in cashmere goats by targeting *WNT10A* and *GNAI2* [35]. Also, miRNA-203 may be involved in regulating hair follicle development in cashmere goats by targeting *NAE1* and *DDOST* [11]. In this study, the GO of the DEmiRNA targets were mainly distributed in cell processes, cell part, binding, single-organism processes, and developmental processes. In addition, miR-211 plays an important role in the formation of black and white skin hair follicles in goats [36], where the expression of miR-211 in melanoma cells is significantly lower compared to normal melanocytes. Interestingly, miR-211 is also identified as a key DEmiRNA in this study, targeting multiple melanogenesis-related genes identified in the research. Research has shown that miR-493-5p knockout significantly changed the role of NR2F1-AS1 in the occurrence and progression of melanoma [37], while the miR-493-3p/HNRNPU/COMT/Dopamine axis may be related to melanocyte imbalance in the pathogenesis of segmental vitiligo [38]. In this work, miR-211, miR-493-3p, miR-493-5p, and miR-379-5p were key DEmiRNAs between groups, indicating that they may also be involved in the hair follicle development and melanogenesis in goats.

Many reports have shown that miRNA can directly target melanogenesis related genes, while lncRNA and circRNA can regulate pigmentation in many ways [31]. In addition, the cceRNA is receiving more research attention. The circRNA-1926/miR-148a/b-3p/CDK19 axis regulates the differentiation of SHF-SCs into hair follicle lineages in cashmere goats [22], while CircRNA-0100 can regulate the differentiation of cashmere goat SHF-SCs into hair follicle lineage by mediating miR-153-3p to change KLF5 expression [21].

In the present study, we focused on the DEcircRNA-DEmiRNA-DEmRNA and DElncRNA-DEmiRNA-DEmRNA co-expression network in the skin of DBGs and IMCGs. The prediction results highlighted a few combinations of importance. For example, chi-miR-211 potentially binds circ_DLG1_1 and *TRPM1*, Capra_hircus newGene_12605 while chi-miR-493-3p binds circSERINC_4, *COL3A1* and *SLC45A2*, and chi-miR-503 binds lncRNA MSTRG.173758.2 and *SLC45A2*. This suggests that DEncRNAs and DEmRNAs may cooperatively regulate hair follicle development and melanogenesis via an interaction network.

Notably, the mRNAs in the co-expression networks were found to be closely associated with the development of hair follicles and pigmentation in goat skin. For example, a number of studies have demonstrated that *DCT* can participate in melanogenesis regulation as a melanocyte related gene [39]. Moreover, transcriptomic analysis of both white and black sheep skin, employing global gene expression profiling, revealed a significant upregulation of *DCT* expression specifically in black sheep skin [40]. *SHISA3* is enriched into the Ras signaling pathway, the MAPK signaling pathway, the PI3K Akt signaling pathway and the Rap1 signaling pathway, to participate in hair length differentiation [41]. Studies have also suggested *TRPM1* participation in melanocyte differentiation and proliferation [42], while transcriptome sequencing of different skin regions in Wuzhishan pigs revealed significantly higher expression of the TRPM1 gene in the black hair of the back compared to the white hair of the abdomen [29]. *SLC45A2* partakes in the transport of melanin synthesis proteins, and it is considered one of the most important genes affecting human pigmentation [43]. Similarly, *COL13A1* can induce globular formation of dermal papillary cells to enhance dermal papilla cell maintenance and hair follicle induction [44]. A recent study confirmed the role of *IGF2BP2* in melanoma development through the MSC-AS1/miR-302a-3p/IGF2BP2/LEF1 axis [45]. Hence, the results from this study, in conjunction with previous work, suggest that these genes may participate in the development of pigmentation and the hair follicle in goat skin through the regulation of the co-expression network. By increasing data available on ncRNAs, we better understand the genetic regulation that may coordinate melanogenesis and hair follicle development in goats. Finally, we anticipate that the discovery of new circRNAs and lncRNAs will promote the understanding of goat hair color and villus development. Novel_miR_741, as the miRNA we identified, interacts with the most nc RNA molecules in the ceRNA network, indicating its potential as a key factor in the ceRNA regulatory network. And circRNAs such as novel_circ_43 and lncRNAs such as MSTRG.122469.11 may regulate melanin production by adsorbing novel_miR741 to target DCT and TRPM1.

Finally, the DEncRNA and DEmRNA co-expression networks were constructed to investigate the relationship between circRNAs and miRNAs and the relationship between lncRNAs and miRNAs in goat skin. The circRNA-miRNA-mRNA co-expression and lncRNA-miRNA-mRNA co-expression networks were constructed to explore the relationship between circRNA, lncRNA, miRNA and mRNA in goat skin. However, ncRNA ceRNA networks needs further study to verify the targeting relationship and expression amount.

## CONCLUSION

The present study provides a comprehensive insight into the distinctions within the whole-transcriptome sequencing profiles of skin tissues between the DBG and the IMCG. In summary, 157 DEmRNAs, 234 DElncRNAs, 72 DEmiRNAs, and 177 circRNAs were identified and found to be enriched in signaling pathways related to hair follicle development and melanogenesis. Several ceRNA networks were established, suggesting their potential key role in regulating hair follicle development and melanogenesis. The results revealed the mRNA and ncRNA profiles in the skin of distinct goat breeds, providing valuable insights for future investigations into the molecular mechanisms governing hair follicle development and melanin synthesis.

## SUPPLEMENTARY MATERIAL

**Supplement 1**. The sequences of all primers.

**Supplement 2**. AllSample_GC_Q. **Supplement 3**. sRNA filter.
