# Supplementary material for: Whole-transcriptome RNA sequencing reveals global expression dynamics and ceRNA regulatory networks related to hair follicle development and melanogenesis in goats
Source: Anim Biosci. 2025 Mar 31;38(9):1841–57. doi: 10.5713/ab.24.0617 (PMC12415471; doi:10.5713/ab.24.0617)
Supplement: Supplementary file 1 [file ab-24-0617-Supplementary-1.pdf]

Supplement 1. The sequences of all primers.

Name	Primers (5′–3′)
TRPM1	F: TGCCTACCGCTGCAACTACAC<br>R: CGGCTCGTCATCTTCCATTCC
DCT	F: GGGATGAGAACTTCACGATT<br>R: TGGGCTGAGCAGATTAGGGT
SLC45A2	F: CAGATCCTGGTCGGAAGTGG<br>R: TGTCTGAGGTTAGGGACCGT
MSTRG.13651.1	F: FCTACCTTCTCCATACTGTGTGATGG<br>R: CTACAGTTCATAAGGTCGCAGAGTC
MSTRG.199158.10	F: GGTGTTGGTTGATATAGACAGCAGG<br>R: CATTTTCAGGGCTAGTTGATTCGG
MSTRG.112838.7	F: CTTCCCATTAGATAGGCGTATCTCC<br>R: CCTCTCTGATGTCACAAAGCTAGAG
circRGL1_1	F: AACCAGAAGCGGACCCAG<br>R: CGGATCGTAGGAGCCAAG
circBICC1_2	F: CAGCTTCTATTGGCAGCCCTAA<br>R: TCTCGGCAGCACGCTCACTC
noval_circ_387	F: GCAAATCAAGAGGTTCAA<br>R: GGGTTTTCTGGGTCAAGT
chi-miR-211	F: TCGGCAGGTCCCTTTGTCATCC<br>R: TGCAGGTCAACTGGTGTCGT<br>Loop: CTCAACTGGTGTCGTGGAGTCGGCAATTCAGTTGAGTGGGCAAA
chi-miR-379-5p	F: GCGCGTGGTAGACTATGGAA<br>R: ATCCAGTGCAGGGTCCGAGG<br>Loop: GTCGTATCCAGTGCAGGGTCCGAGGTATTCGCACTGGATACGACCCTACG
chi-miR-411a-5p	F: CGCGCGATAGTAGACCGTATA<br>R: ATCCAGTGCAGGGTCCGAGG<br>Loop: GTCGTATCCAGTGCAGGGTCCGAGGTATTCGCACTGGATACGACGTACGC
chi-miR-493-3p	F: AAGCGACCTGAAGGTCTACTGTG<br>R: ATCCAGTGCAGGGTCCGAGG<br>Loop: GTCGTATCCAGTGCAGGGTCCGAGGTATTCGCACTGGATACGACCCTGGC
β -actin	F: CACTCTTCCAGCCTTCCTTC<br>R: GTACAGGTCTTTGCGGATGT
U6	Forward: CTCGCTTCGGCAGCACA<br>Reverse: AACGCTTCACGAATTTGCGT
