# Supplementary material for: Whole-transcriptome RNA sequencing reveals global expression dynamics and ceRNA regulatory networks related to hair follicle development and melanogenesis in goats
Source: Anim Biosci. 2025 Mar 31;38(9):1841–57. doi: 10.5713/ab.24.0617 (PMC12415471; doi:10.5713/ab.24.0617)
Supplement: Supplementary file 2 [file ab-24-0617-Supplementary-2,3.pdf]

Supplement 2. AllSample_GC_Q

#SampleID	ReadSum	BaseSum	GC(%)	N(%)	Q20(%)	Q30(%)
DBG1	55668409	16367409886	48.94	0.00	98.14	94.61
DBG2	51889577	15350741850	49.48	0.00	98.10	94.58
DBG3	51778867	15256878826	49.06	0.00	98.07	94.57
IMCG1	52166317	15486050040	49.03	0.00	97.96	94.17
IMCG2	51839903	15287152768	49.86	0.00	98.06	94.49
IMCG3	56053723	16557225902	49.39	0.00	98.25	94.93

Supplement 3. sRNA filter

Samples	BMK-ID	Raw_reads	Low_quality	Containing'N'reads	Length<18	Length>30	Clean_reads	Q30(%)
DBG1	DBG1	34251481	0	0	495025	1490739	32265717	95.20
DBG2	DBG2	22769494	0	0	596988	1506060	20666446	96.54
DBG3	DBG3	23523225	0	0	531158	2199874	20792193	96.68
IMCG1	IMCG1	22756677	0	0	610955	1208152	20937570	96.62
IMCG2	IMCG2	314833110	0	583262	753213	30146836	95.81	
IMCG3	IMCG3	25710955	0	0	1971888	925876	22813191	96.75
